# Radiative impact of record-breaking wildfires from integrated ground-based data

**DOI:** 10.1038/s41598-025-85103-1

**Published:** 2025-03-10

**Authors:** Evgueni Kassianov, Connor J. Flynn, James C. Barnard, Larry K. Berg, Sherman J. Beus, Xingyuan Chen, Swarup China, Jennifer M. Comstock, Brian D. Ermold, Abdulamid A. Fakoya, Gourihar Kulkarni, Nurun Nahar Lata, Nate G. Mcdowell, Victor R. Morris, Mikhail S. Pekour, Hans J. Rasmussen, Laura D. Riihimaki, Mingjie Shi, Manish Shrivastava, Hagen Telg, Alla Zelenyuk, Damao Zhang

**Affiliations:** 1 Pacific Northwest National Laboratory, Richland, WA USA; 2 School of Meteorology, University of Oklahoma, Norman, OK USA; 3 University of Nevada, Reno, NV USA; 4 School of Biological Sciences, Washington State University, Pullman, WA USA; 5 Cooperative Institute for Research in the Environmental Sciences, Boulder, CO USA; 6 Global Monitoring Laboratory, National Oceanic and Atmospheric Administration, Boulder, CO USA

**Keywords:** Climate sciences, Ecology, Environmental sciences, Hydrology, Natural hazards

## Abstract

The radiative effects of wildfires have been traditionally estimated by models using radiative transfer calculations. Assessment of model-predicted radiative effects commonly involves information on observation-based aerosol optical properties. However, lack or incompleteness of this information for dense plumes generated by intense wildfires reduces substantially the applicability of this assessment. Here we introduce a novel method that provides additional observational constraints for such assessments using widely available ground-based measurements of shortwave and spectrally resolved irradiances and aerosol optical depth (AOD) in the visible and near-infrared spectral ranges. We apply our method to quantify the radiative impact of the record-breaking wildfires that occurred in the Western US in September 2020. For our quantification we use integrated ground-based data collected at the Atmospheric Measurements Laboratory in Richland, Washington, USA with a location frequently downwind of wildfires in the Western US. We demonstrate that remarkably dense plumes generated by these wildfires strongly reduced the solar surface irradiance (up to 70% or 450 Wm^-2^ for total shortwave flux) and almost completely masked the sun from view due to extremely large AOD (above 10 at 500 nm wavelength). We also demonstrate that the plume-induced radiative impact is comparable in magnitude with those produced by a violent volcano eruption occurred in the Western US in 1980 and continental cumuli.

## Introduction

Wildfires are now frequently in the forefront of the news as one of the most impactful natural hazards and disturbances of ecosystems^1,2^. Wildfire events owe their existence to an interactive coupling between strong winds, high temperatures, and moisture supply deficit for fire-prone regions. These events represent a significant source of gaseous and particulate emissions at both regional and global scales^3–5^. Fire emissions have a considerable influence on solar energy production^6^, biogeochemical and hydrological cycles^7^, atmospheric chemistry and composition^8,9^, as well as air quality and human health^10–12^. The emission characteristics determine biomass burning (BB) optical properties with pronounced temporal and spatial changes due to BB plume evolution and mixing with other atmospheric species^13^. These co-occurring evolution and mixing processes are persistent challenges for both modeling and observational studies. Integrated measurements from surface, air, and space are essential components that are needed to properly capture these complex processes^14–16^ and to assess their impact on the radiation budget^17,18^.

State-of-the-art models, such as the Weather Research and Forecasting (WRF) model coupled with Chemistry (WRF-Chem), are commonly employed to simulate the wildfire events and their radiative effect across various spatial and temporal scales^19,20^. These models calculate aerosol extinction efficiency, vertically resolved extinction coefficient, single scattering albedo (SSA), and asymmetry parameter (ASY) or their columnar integrated counterparts using refractive indices in each internally mixed aerosol size bin and wavelength. Traditionally, assessment of model predictions involves observation-based aerosol optical properties, such as aerosol optical depth (AOD), provided by ground-based measurements and/or satellite retrievals^19,21^. However, the frequent lack of these properties for the dense BB plumes due to measurement limits^22,23^ prevents a “traditional” assessment. On the other hand, these optical properties calculated for a model-specified mixture and size distribution of aerosol together with surface albedo computed for an assumed surface type are used for calculations of radiative properties, including downwelling surface irradiance, over shortwave (SW)^24^ and longwave^25^ spectrum as documented, for example, by Liu et al., 2020^19^.

The radiative effect of the wildfire events is typically defined as a difference between “polluted” (with BB plume) and “clean” (without BB plume) radiative properties predicted by a model for a given time and location^26,27^. Thus, an assessment of model-predicted radiative effects could be performed if their observational constraints are available. Many ground-based sites around the world provide observations of downwelling surface irradiance^28–30^. Moreover, these observations have been used to estimate the radiative effects of clouds by a conventional method^31^. Dense plumes generated by intense wildfires are frequently characterized by exceptionally high AOD values (above 7) at visible wavelengths^22^, which resemble optical thickness of several cloud types, such as continental cumuli^32^. Recall, cloud optical thickness (COT) defines the overall attenuation of downwelling solar radiation by cloud droplets in the atmospheric column. The outlined resemblance between AOD and COT values leaves one wondering whether this conventional method^31^ could be extended successfully for assessing the radiative effects of intense BB events. Our paper answers this important question by introducing a new method and illustrating its performance for intense BB events occurred in the Western US in September 2020.

In the following sections, we describe several pathways for detecting plumes associated with the record-breaking wildfires, quantify the plume-induced radiative effects using our method, and compare them with those produced by a violent volcano eruption and continental cumuli (“Results” section). Then, our paper underscores the challenges tied to the limited availability of observation-based aerosol optical properties for dense plumes, highlights the significance of our results, and outlines our method, along with its anticipated broad practical applications in a variety of climate-related areas (“Discussion” section). The paper concludes by offering a detailed description of our approach, emphasizing its novelty and flexibility (“Method” section).

## Results

### Specified duration and area

**Fig. 1:**
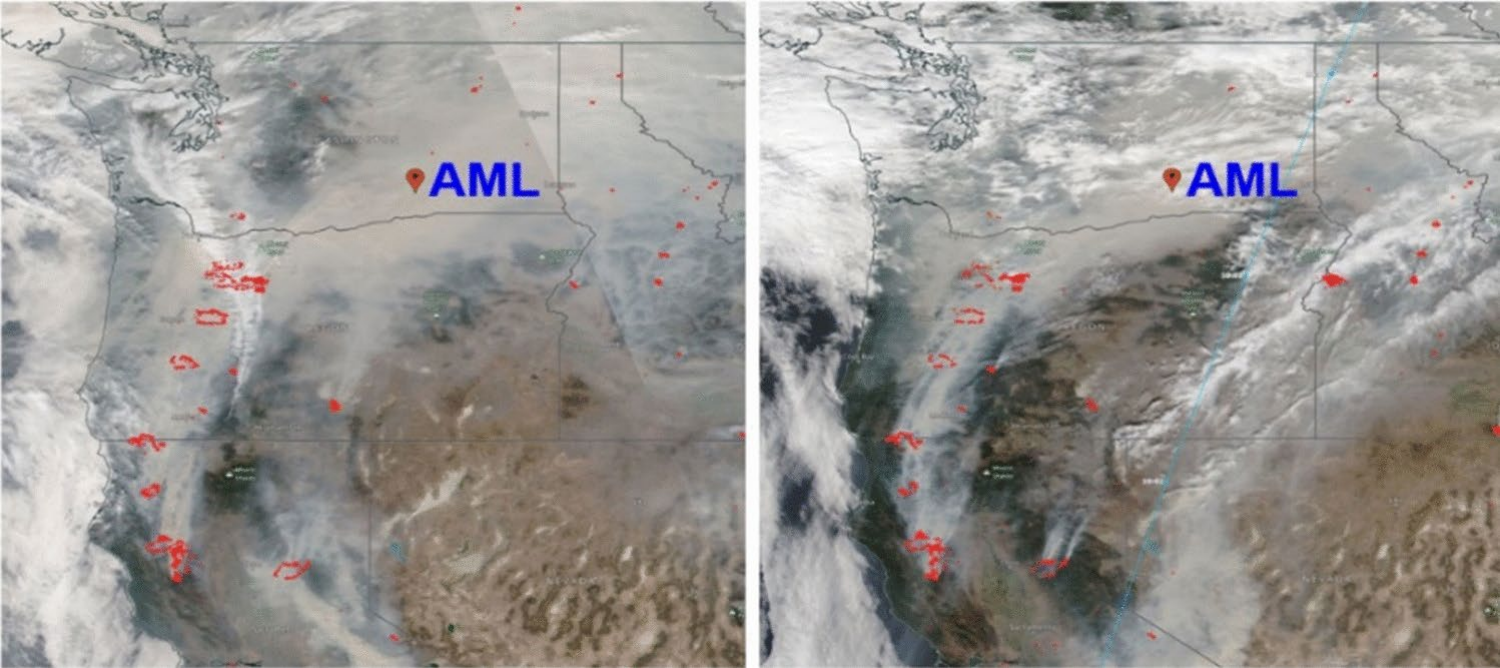
These NASA Aqua satellite images show smoke plumes (grey) and clouds (white swathes) in USA’s Pacific Northwest on September 13 (left) and September 14 (right), 2020. The plumes originated from numerous wildfires (red dots) over three states and moved northeastward over the AML (red marker) due to a strong wind surge in the easterlies. The images were generated using the NASA EOSDIS Worldview version 3.25 (https://worldview.earthdata.nasa.gov/).

September 2020 was distinguished by remarkably impactful wildfires which occurred in three western US states (California, Oregon, and Washington). These wildfires were a source of fascination for the public mind and became a “hot” topic for researchers^33–35^. Strong winds associated with highly unusual synoptic environment^36^ picked up the BB particles, injected them into the atmosphere at elevated altitudes and then carried them northeastward across the US over 19 states during September 14-17^th^, 2020^34^. For our case study, we use data collected by a suite of ground-based instruments deployed at the Atmospheric Measurements Laboratory (AML) located in Richland, Washington (WA) state (46.341°N, 119.279°W) (Fig. 1). We select a 12-day period (September 9-20^th^, 2020), which includes several “clean” days observed at the AML both before (September 9-10^th^, 2020) and after (September 19-20^th^, 2020) the “polluted” episode (“Different ways of detecting BB events” section). Data collected during “clean” days are imperative for our approach (“Method” section) with expected applications (“Discussion” section).

Synthesizing measurements from different sources could enhance our understanding of the complexity and strong non-stationarity of smoke plumes. Satellite-based studies have offered valuable information about plume top heights and aerosol loading aloft at regular time intervals^35^. However, plume base heights and their diurnal changes, which are strongly relevant to health issues, have rarely been provided by orbital sensors, especially for optically dense plumes. Ground-based instruments, such as ceilometers, can greatly complement the orbital sensors and deliver plume base heights with a high temporal resolution (2 s). The AML instrumental suite (**Supplementary information S1**) includes a ceilometer, radiometers for measuring both broadband and narrowband downwelling surface irradiances, and a CIMEL sunphotometer (CSPHOT) for measuring/retrieving aerosol columnar properties, such as AOD, SSA and ASY. Small and large values of AOD denote “clean” and “polluted” conditions, respectively. Assessment of the spatial representativeness of the AML AOD under “clean” and “polluted” conditions is performed using satellite data (**Supplementary information S2**). Valuable characterization of near-surface ambient aerosol particles during these conditions (**Supplementary information S3**) was conducted at the AML and the co-located Environmental Molecular Sciences Laboratory (EMSL), a national scientific user facility sponsored by the U.S. Department of Energy’s (U.S. DOE) Office of Biological and Environmental Research. The AML and EMSL are part of the Pacific Northwest National Laboratory (PNNL) campus.

The predominant downwind location (Fig. 1) and variety of commonly used ground-based instruments make the AML particularly appealing for quantifying the impact of smoke plumes on surface solar radiation for different burdens and vertical distributions of BB aerosols. It should be emphasized that the AML instruments, data collection and processing are identical to those supported by the Atmospheric Radiation Measurement (ARM) user facility (https://www.arm.gov). Since the ARM instruments are well-documented, we include only a short summary with the corresponding references (**Supplementary information S1**). Consequently, we use the “ARM-like” data collected at the AML for our study.

### Different ways of detecting BB events

**Fig. 2:**
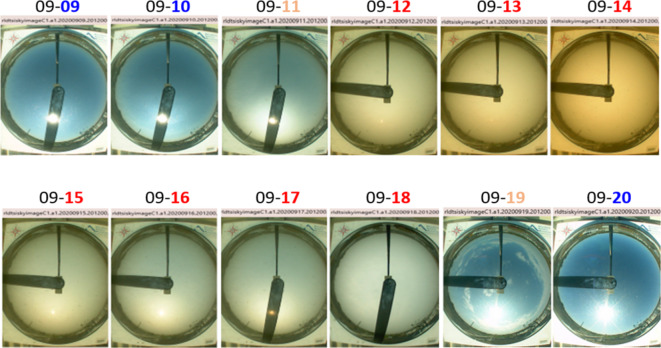
These ground-based images provided by the AML Total Sky Imager (TSI) show large changes of the sky from blue (“clean” days) to grey-orange (“polluted” days) and then back to blue (“clean” days) during the selected period at the same time (20:12:00 UTC).

Here we illustrate the application of integrated ground-based measurements to detect the BB plumes and capture their evolution. A visual representation of such evolution is provided by ground-based images (Fig. 2) supplied by the AML Total Sky Imager (TSI). This instrument is set up to scan the same portion of sky at an interval of 30 s, mimicking what a surface observer would do at the same location. Both the sky color (blue to grey to orange) and its darkness (ability to see the sun clearly – or not) are visual clues of the presence and thickness of a plume. These clues suggest that the leading edge of the plume appears over the AML on the third day (September 11, 2020) of the selected period (Fig. 2). In turn, striking “Mars-like” grey-orange color of the sky, making the sun invisible during the next three “dark” days (September 12–14, 2020), signifies the presence of a dense plume over the AML. Finally, the blue sky with bright sun seen on the last two days (September 19 and 20, 2020) indicates that the plume has moved out of the area. It is important to emphasize that the TSI images (Fig. 2) are included for *qualitative* value only, while “polluted” and “clean” periods are defined *quantitatively* using combination of data, such as AODs, backscatter profiles, and surface irradiances, offered by several ground-based instruments. Application of these data for detection of plumes and capturing their evolution is discussed below.

**Fig. 3:**
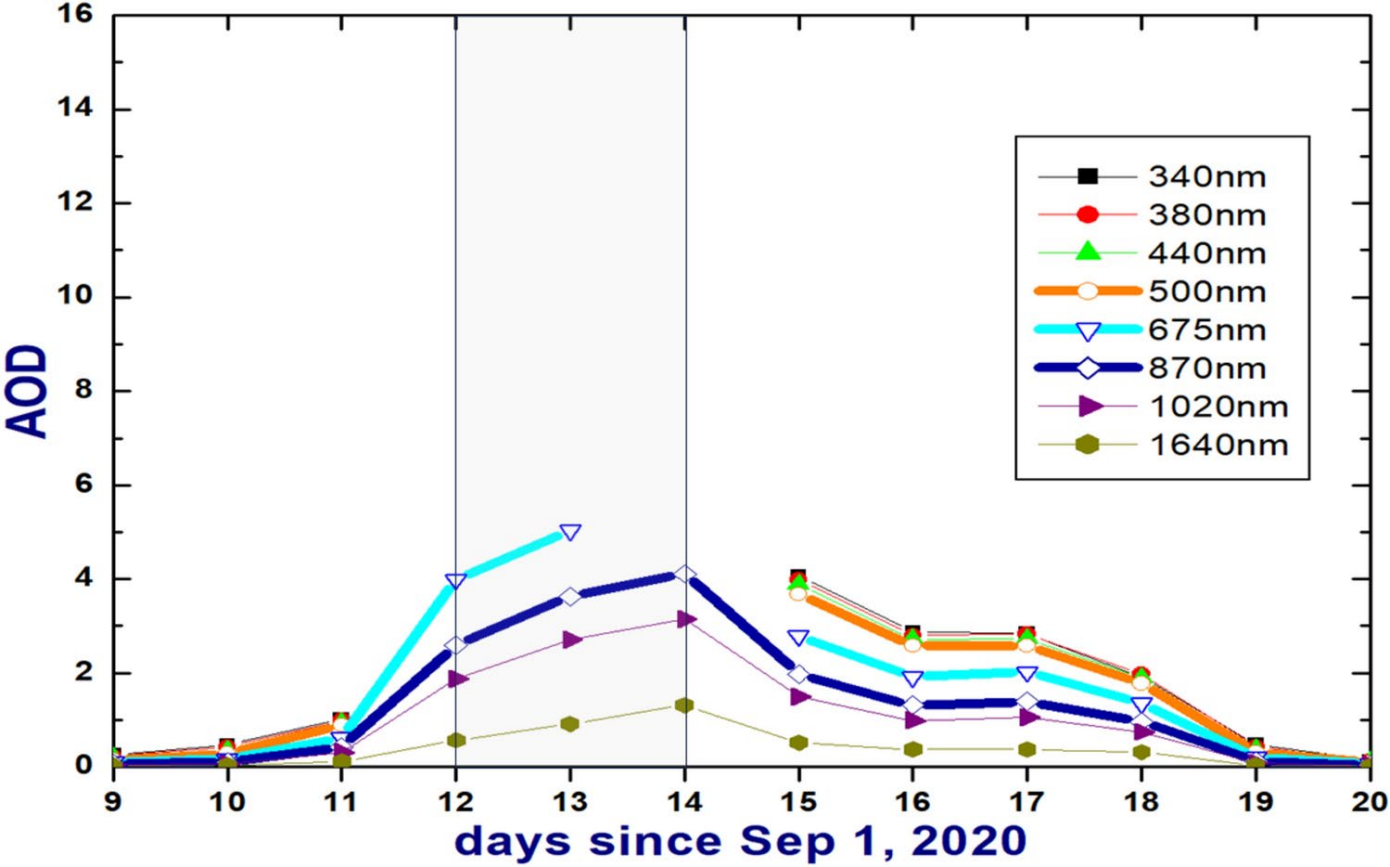
Diurnal changes of AOD measured by AML CSPHOT at eight wavelengths (340, 380, 440, 500, 675, 870, 1020 and 1640 nm) during the selected period. The grey box indicates three days (September 12,13 and 14, 2020) where AOD values measured at shorter wavelengths are not available.

There are remarkable signatures of the heavy smoke in spectrally resolved AODs measured by the AML CSPHOT (Fig. 3). Recall, the AOD represents total aerosol burden in the atmospheric column. The AOD measurements hinge on the fact that the direct beam signal becomes smaller as the AOD increases. Thus, the smallest signal (comparable with the measurement uncertainty) occurs when aerosol loading is the largest due to nearly complete attenuation of direct solar radiation reaching the surface. The upper limit of AOD measured by CSPHOT at a given wavelength is assigned as AOD(λ)*m < 7^22^, where *m* is optical air mass roughly inversely proportional to cosine of solar zenith angle and λ is wavelength. The AOD measured by the CSPHOT at shorter wavelengths (less than 675 nm) during these “dark” days (September 12–14, 2020) surpass the upper measurement limit, and thus these AODs are not available (Fig. 3). A similar inability to measure AODs at shorter wavelengths was documented for the massive Indonesian biomass burning episode^22^. Note that the AML AODs are quite representative for a large area (roughly 100 × 100 km) when aerosol loading is small-to-moderate (AOD at 550 nm is smaller than 0.5) (Fig. S3).

**Fig. 4:**
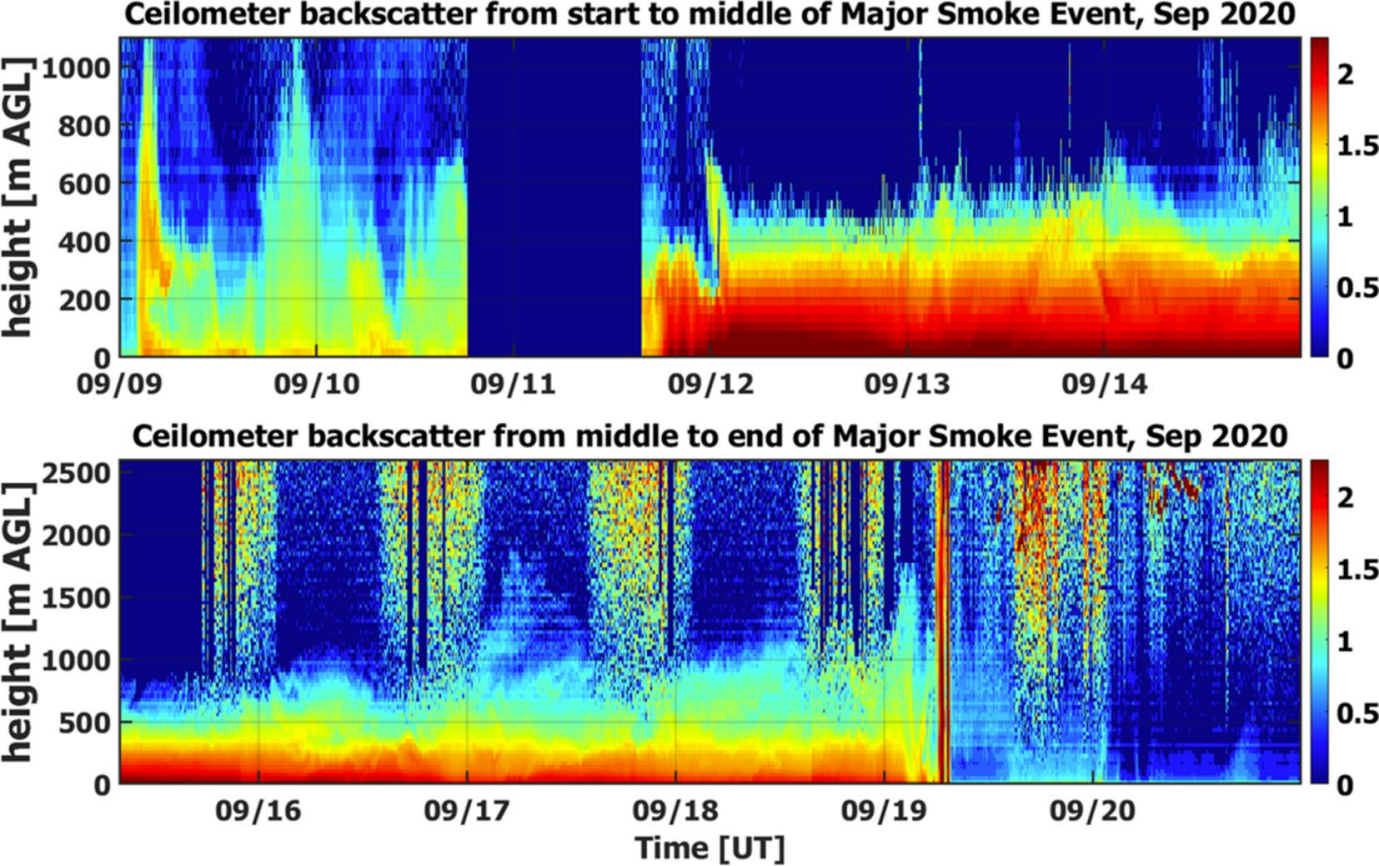
Time–height images of attenuated backscatter (10^–4^ srad^-1^ km^-1^) derived from the AML ceilometer for two sub-periods: from September 09, 2020 to September 14, 2020 (the first sub-period; top panel) and from September 15, 2020 to September 20, 2020 (the second sub-period; bottom panel). In both panels, horizontal axis is time (calendar date in September 2020, UTC) and vertical axis is altitude (m). Red color represents strong backscatter signal on a log10-scale, indicating a high concentration of aerosol. Conversely, blue color denotes the opposite due to low aerosol loading or strongly attenuated signal. In the top panel, the blue vertical stripe on September 11, 2020, defines missing data.

While the satellite measurements give limited information on the vertical distribution of the dense BB plumes with high temporal resolution, the AML ceilometer offers such valuable information near the surface (Fig. 4). In particular, the backscatter images provided by the AML ceilometer indicate clearly that the largest values of the backscatter signal occurred at ground level. In other words, the lower edge of BB plume detected by the ceilometer was almost touching the ground. The observed high concentration of particles near the surface (Fig. 4) caused very unhealthy and hazardous air quality in Richland, WA for several days (September 12–15, 2020). Both sub- and supermicron particles contribute to these harmful conditions (Figs. S4-S7). Likely, the BB plume has a vertical structure, which the ceilometer is unable to detect due to the strong signal attenuation.

**Fig. 5:**
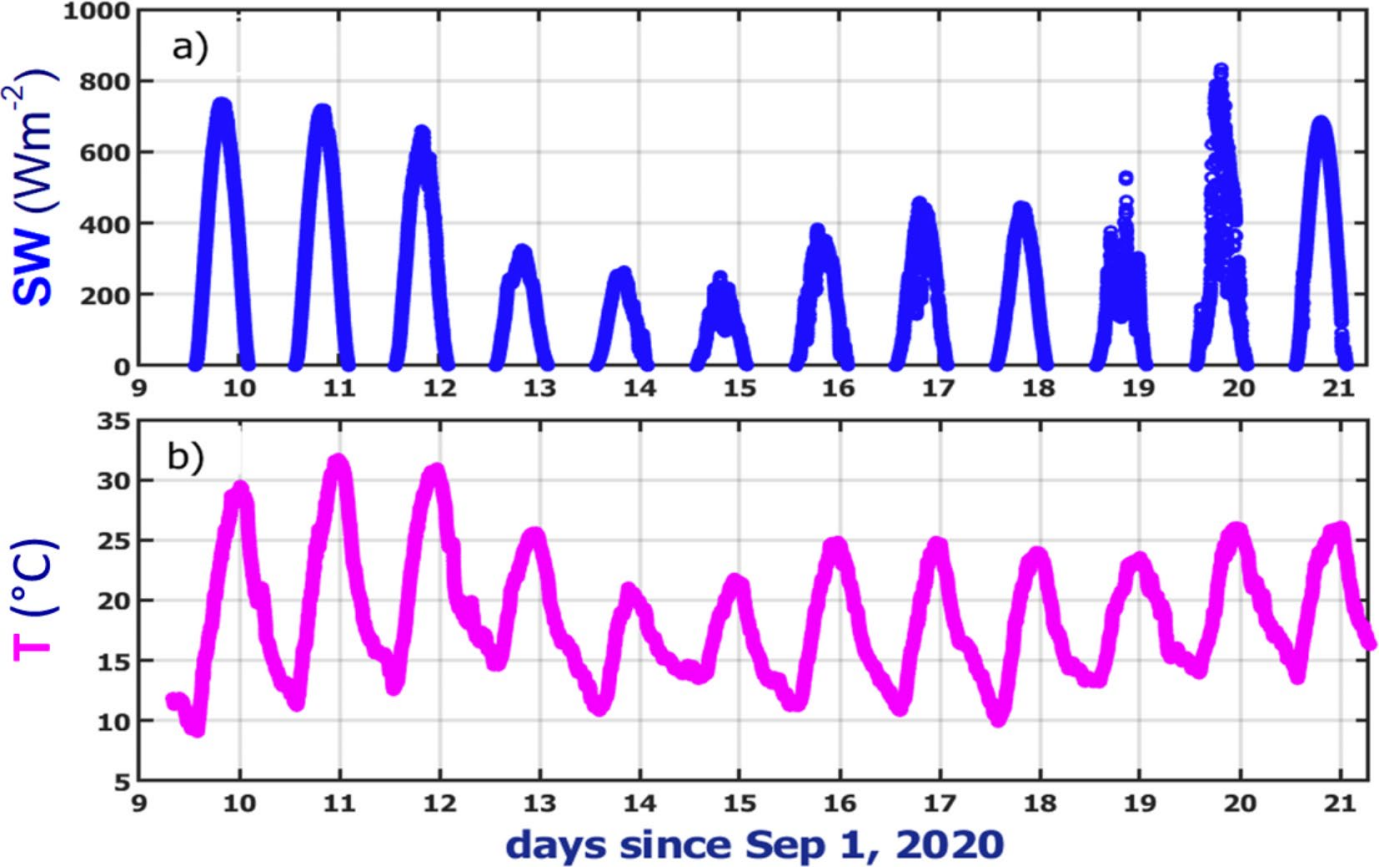
Temporal variability of the total SW irradiance (a) and air temperature (b) measured at the AML during the selected period. Horizontal axis is time (calendar date in September 2020, UTC).

The smallest values of SW irradiance (Fig. 5a) occur for the “darkest” day (September 14, 2020) in comparison with those measured for cleaner days in terms of the daily-averaged AOD (Fig. 3). For example, the SW irradiance measured near the local noon is about 700 Wm^-2^ and 300 Wm^-2^ for “clean” (September 20, 2020) and the “darkest” (September 14, 2020) day, respectively. The corresponding daily-averaged values of AOD measured at 870 nm wavelength are about 0.04 and 4.1, respectively (Fig. 3). To put it differently, the strong temporal variations of the aerosol loading is mostly responsible for substantial attenuation of the SW solar radiation reaching the surface around the local noon. Such attenuation results in considerable reduction of air temperature (Fig. 5b) due to a lack of surface heating by the sun. Consequently, the maximum air temperature measured near the local noon is reduced by about 5 °C (roughly from 31 °C to 26 °C) during a 24-h period (September 11–12, 2020; Fig. 5b), which represents a transition from “clean” to “dark” observational conditions.

### Different ways of assessing BB events

**Fig. 6:**
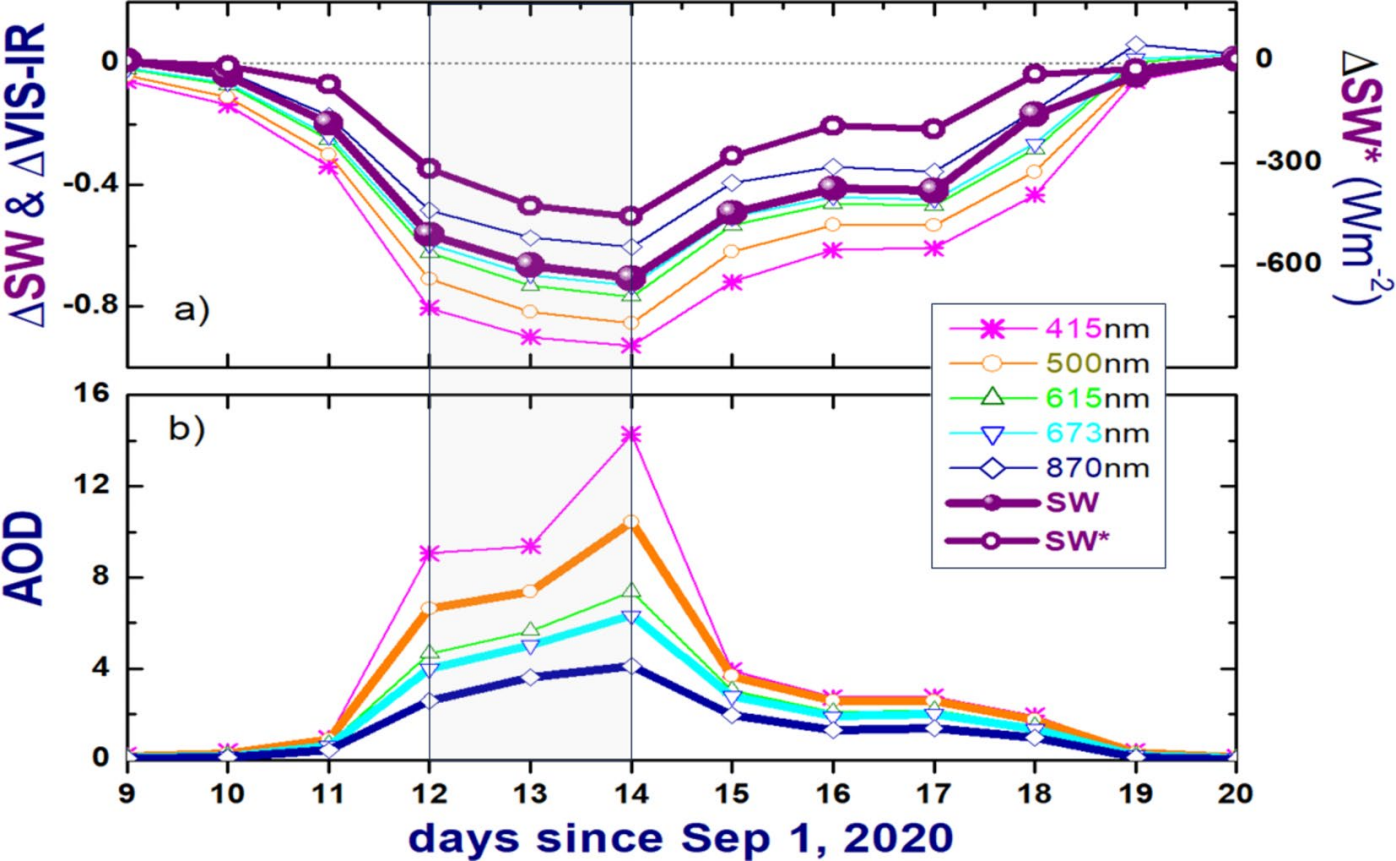
Day-to-day changes of the calculated non-normalized (purple; symbol ΔSW*) and normalized (purple; symbol ΔSW) difference of the SW irradiance and its five spectrally resolved normalized components (symbol ΔVIS-IR) at five wavelengths (415, 500, 615, 673 and 870nm) in the visible (VIS) and near-infrared (IR) spectral range during the selected period (a). The corresponding changes of five spectrally resolved AODs (b). See text for details.

The normalized difference between the measured “polluted” and estimated “clean” irradiances provides a quantitative assessment of smoke-induced attenuation of solar radiation reaching the surface (Fig. 6a). For the total SW irradiance, the normalized difference (ΔSW = (SW_polluted_-SW_clean_)/SW_clean_) reduces steadily, dips (about—0.7) for the “darkest” day (September 14, 2020) and then increases gradually until it reaches the maximum on the “clean” day (September 20, 2020). The largest magnitude (about -0.7) of the ΔSW indicates that the dense smoke plume can strongly (up to 70%) reduce the total SW surface irradiance in comparison with its counterpart that would be measured for a given day without the presence of BB plume. Similar reduction (~ 70%) of the total SW surface irradiance was produced by a single-layer continental cumuli observed at the ARM site in Oklahoma state on July 28, 2007^37^. Commonly, these clouds have moderate (~ 10) values of cloud optical depth^32^, which are generally coincide with AODs estimated for the “dark” days (e.g., September 13 and 14, 2020). The largest magnitude (about -0.7) of the ΔSW (Fig. 6a) represents the corresponding largest magnitude (about -450 Wm^-2^) of its non-normalized counterpart (ΔSW* = SW_polluted_-SW_clean_) (Fig. 6a). Note that averaged values of the ΔSW and ΔSW* (Fig. 6a) are calculated for instances with cloud-screened and quality-assured AODs for a given day (“Method” section).

The plume-induced reduction is even more substantial (up to 93%) for the spectral components (ΔVIS-IR(λ) = (SW(λ)_polluted_-SW(λ)_clean_)/SW(λ)_clean_) of the total SW irradiance, which describe five wavelengths (415, 500, 615, 673 and 870 nm) located within the VIS and near-IR spectral range (Fig. 6a). These spectral components (Fig. S1) are measured by a Multifilter Rotating Shadowband Radiometer (MFRSR) collocated with both broadband radiometer and CSPHOT at the AML. The day-to-day changes of the ΔVIS-IR have a “U-shape” (Fig. 6a) and mirror those obtained for the spectrally resolved AODs (Fig. 6b). The latter define both available CSPHOT-measured AODs and AODs calculated using the Ångström exponents (AEs) of the CSPHOT-measured AODs similar to Eck et al., 2019^22^. For the “clean” day (September 20, 2020), for example, the CSPHOT-measured AODs are available at eight wavelengths (340, 380, 440, 500, 675, 870, 1020, 1640 nm). Thus, the 380- to 440nm AE is used to for calculation of AOD at 415nm wavelength, while the 500- to 675nm AE is used to calculate AOD at 615nm wavelength. For the “darkest” day (September 14, 2020), the CSPHOT-measured AODs are available at three wavelengths (870, 1020, 1640 nm) only (Fig. 3). Thus, the 870- to 1020nm AE is used for calculation of AOD at four wavelengths (415, 500, 615, 675 nm). Since the accuracy of the outlined AE-based calculations of the spectrally resolved AOD is unknown, such calculations should be used with caution.

**Fig. 7:**
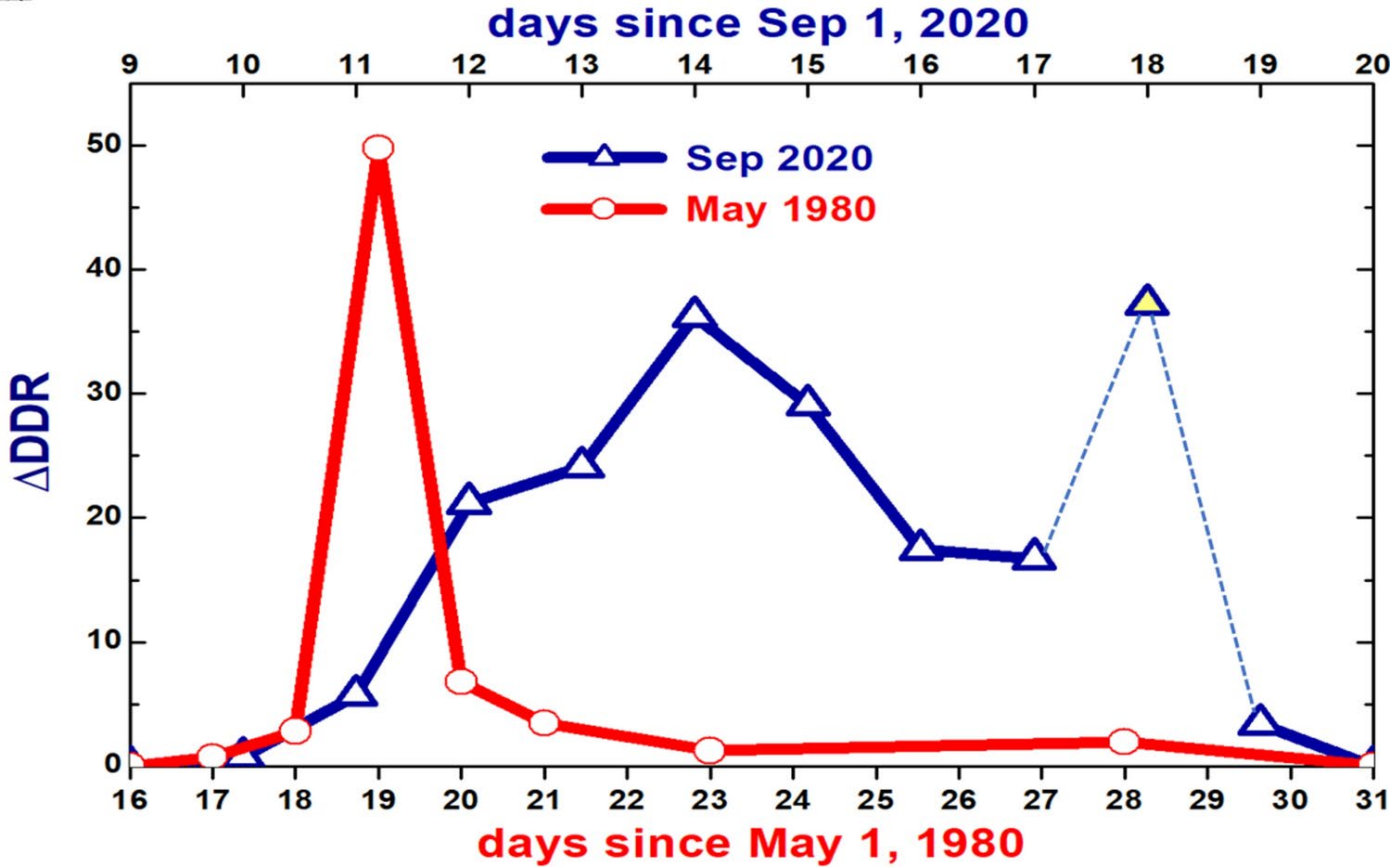
Day-to-day changes of the calculated normalized difference of the diffuse-to-direct ratio (ΔDDR) for two periods: from May 16, 1980 to May 31, 1980 (the first “volcano” period; red) and from September 9, 2020 to September 20, 2020 (the second “BB” period; blue). The DDRs measured at the HMS in the morning (8am)^38^ are used for the first period, while both diffuse and direct components of the total SW irradiance measured at the AML are used for the second period. The calculated ΔDDR for a cloudy day (September 18, 2020) indicates a substantial impact of cloud contamination on the calculated ΔDDR. This cloud contamination is highlighted by a symbol filled with yellow color and thin dotted lines.

For the sake of comparison, we also apply our approach to illustrate the impact of a giant plume produced by a volcano (Mount St. Helens; WA) during its disastrous eruption (May 18, 1980). The volcano plume covered a substantial area of three downwind US states (WA, Idaho, and Montana) during its long-range eastern travel and swept over Richland, WA located about 200 km from Mount St. Helens. The SW surface irradiance has been measured before, during and after this eruption by a ground-based radiometer temporally deployed at the Hanford Meteorological Station (HMS)^38^, located approximately 35 km northwest of the AML and equipped with a similar radiometer as in the AML instrumental suite for measuring the SW surface irradiance. Here we illustrate application of our approach to the ratio of diffuse and direct components of the total SW irradiance, the so-called Diffuse-to-Direct Ratio (DDR)^38^. To do that, we calculate the normalized difference ΔDDR between “polluted” and “clean” values of DDR (Fig. 7) using “clean” DDR for normalization and assuming that May 16, 1980 and September 20, 2020 represent “clean” days.

The DDR calculated at the HMS has a huge peak (about 50 times relative to “clean” day) on the morning of May 19, 1980 after the volcano eruption (Fig. 7). Thin cirrus clouds apparently occurred during this day^38^, which could have contributed to this DDR peak. The ΔDDR calculated at the AML has a comparable peak (about 40 times relative to “clean” day) on the “darkest” day (September 14, 2020) (Fig. 7). Thus, the impacts of the BB (September 14, 2020) and volcano (May 19, 1980) plumes are roughly equal in terms of the ΔDDR magnitude. Compared to the volcano plume, the BB-induced plume has a longer duration and a more prolonged impact on the ΔDDR (several days in September, 2020 versus one day in May, 1980). The strong reduction (about 30 times) of the direct component (Fig. S2a) for periods with large AOD values mostly explains these remarkable peaks of the ΔDDR (Fig. 7). Cloud contamination of the diffuse irradiance on a cloudy day (September 18, 2020) is mostly responsible for the corresponding peak of the ΔDDR (Fig. 7). This peak indicates limitations of the cloud screening based on the measured AOD only for our initial ΔDDR calculations. Improved ΔDDR calculations in our future studies will involve a complimentary analysis of variability of the diffuse surface irradiance measured with a high temporal resolution similar to that suggested by Long and Ackerman^31^.

## Discussion

### Existing challenges

Intensive wildfires occurred over the western US states in 2020, and these wildfire events produced extremely dense BB plumes with exceptionally large AOD values, which can substantially exceed the upper measurement limit of instruments which infer AOD. For example, a very large AOD (about 14) is estimated at 415 nm wavelength for one of the “dark” days (September 14, 2020; Fig. 6b) using available Ångström exponents obtained at longer wavelengths (870 and 1020 nm) and the customary procedure^22^. However, the accuracy and uncertainty of such estimation is unknown. Moreover, other aerosol optical properties, such as columnar SSA and ASY, are also missing even using the most advanced aerosol inversions (Version 3) for days with extremely dense plumes. Thus, three major observation-based aerosol optical properties of these dense plumes are either estimated with unknown uncertainty (AOD; visible spectral range) or little availability (such as SSA and ASY). Potential application of near-surface measurements of the corresponding aerosol optical properties for estimation their columnar equivalents would be questionable due to unknown vertical distribution of a BB plume. As a result, the traditional assessment of model-predicted radiative effects of intensive wildfires, based on observation-derived aerosol optical properties (AOD, SSA, ASY), has limited potential. It is expected that occurrences of these wildfires will increase in intensity and frequency due to rising temperatures and dryness in fire-prone regions^39–42^.

### Novel approach and major finding

A strong need to side-step the long-standing and complex challenges associated with the uncertainties and scarcity of observation-based aerosol optical properties highlighted above motivates us to tailor the conventional method^31^ to provide additional observational constraints for such assessments. Following the conventional method^31^, our approach quantifies the radiative effects of dense plume by comparing the observed “polluted” radiative properties, such as total SW and spectrally resolved irradiances, with their estimated “clean” counterparts. To help with such “clean” estimation, supplementary AOD data are used. To eliminate potential observational uncertainties, calibration issues, effects of solar zenith angle and surface albedo, and to ease comparison with model predictions, our approach implements a normalization. Specifically, the radiative impact of BB plume for a “polluted” day is quantified as a normalized difference between the measured “polluted” radiative property and its estimated “clean” equivalent. The latter is used for the normalization. Our findings show that the radiative effects of the dense BB plumes match roughly those of a major volcanic eruption that occurred in the Western US in 1980 and typical continental cumuli. To illustrate, the dense BB plumes cause a substantial reduction in total SW solar radiation reaching the surface (up to 70% or 450 Wm^-2^), resulting in a significant drop in the maximum air temperature (up to 5 °C) over a 24-h period, making the transition from “clean” to “polluted” conditions. The important consequences of these reductions are outlined in the following section. While our technique has been applied initially to the specific extreme events associated with the record-breaking wildfires and the major volcanic eruption, this technique itself has broad applicability highlighted below.

### Expected applications of novel approach

Our approach can provide important observational constraints for the theoretical underpinning of weather forecasting models, such as the WRF-Chem, using available long-term measurements of the combined AOD and solar radiation^28–30^. Models can predict both SW and spectrally resolved irradiances for dense BB plumes using various configurations, emission inventories, and assumptions regarding evolution and mixing processes. These predicted irradiances can then be used to quantify the radiative effects of the dense BB plumes. Ultimately, the model-based quantifications can be compared with the observational constraints provided by our method. This comparison could assist in evaluating the impact of emission uncertainties and model assumptions on the radiative effects of dense BB plumes. For example, the spectral behavior of the radiative properties depends strongly on relative contributions of different types of sunlight-absorbing aerosols^43^, such as black carbon and brown carbon, with distinct wavelength dependence of light absorption at near-ultraviolet (near-UV), VIS and near-IR wavelengths^44–46^. The spectrally resolved (near-UV, VIS, near-IR) measurements of the solar radiation linked to optical properties of absorbing aerosols can contribute to improved understanding of their radiative effects which remain highly uncertain. Moreover, our approach in conjunction with widely available solar radiation and AOD data^28–30^ can be used for quantification of the radiative effects of optically thick plumes produced by dust storms^47^ and volcanic eruptions^48^.

The quantification highlighted above paves the way for bridging the connection between BB emissions and their ecosystem-related footprints caused by wildfire disturbances. There are important aspects of such connection: (1) the surface energy budget and (2) the partitioning of total downward radiation into its direct and diffuse components. The first three examples are related to the surface energy budget. A recent analysis of integrated radiation, aerosol, and meteorological data collected over the US West Coast during the most destructive wildfire events (September 2020) reveals that the well-defined decrease in near-surface air temperature associated with strong attenuation of sunlight by dense BB plumes suppresses the development of the planetary boundary layer^49^. Additionally, the surface cooling caused by dense BB plumes may contribute to the dissipation of daytime clouds and the suppression of convective precipitation^50^, which in turn could affect synoptic-scale variability of wildfires through intricate wildfire-weather interactions^49^. Moreover, by blocking solar radiation from reaching the surface, dense BB plumes could have a significant impact on solar power generation, resulting in a considerable reduction (up to 65%) in hourly photovoltaic energy production^51^. The following examples are related to the partitioning of total downward radiation. Diffuse radiation is more likely to be absorbed by normally shaded leaves and thus promotes photosynthesis^52,53^. Wildfire-induced AOD increase can raise substantially the fraction of diffuse radiation in the total downward radiation (Figs. S1,S2). The strong sensitivity of vegetation photosynthesis to solar radiation in the VIS spectral range^54^ highlights the expected advantages of spectrally resolved measurements of incoming solar radiation reaching to the surface. Thus, these measurements can be used to quantify vegetation responses to the partitioning of the direct and diffuse radiation over varied landscape types. Knowledge regarding the sensitivity of vegetation productivity to such partitioning can be broadly applied to the state-of-the-art terrestrial biosphere models for improvement of model parameterization and process-based enhancements^52^.

The expected applications of the current version of our approach may not be ideal for selecting “polluted” periods specifically linked to intense BB events and for identifying the corresponding “clear-sky” and “clean” episodes for a given time and location. The corresponding challenges could mainly arise from (1) natural factors, such as the potential presence of thin cirrus clouds and elevated tropospheric or stratospheric aerosols during an anticipated “polluted” period, and (2) observational limitations related to the availability and data quality of combined solar radiation and AOD data. To address these challenges, at least in part, additional efforts are needed.

## Method

There are three major steps of our approach similar to those used in the conventional method^31^: (1) selection of both “clear-sky” (mostly non-cloudy) and “clean” (likely without smoke plume) day within a given period, (2) empirical fitting of the irradiance measured at surface for the selected day and obtaining the corresponding coefficients, and (3) application of these coefficients for estimating a *hypothetical* “clear-sky” and “clean” surface irradiance for a nearby “polluted” day. Distinctions between measured “polluted” and the *hypothetical* “clean” irradiances define the impact of the smoke plumes on the solar radiation reaching the surface. Below we consider mostly total (direct plus diffuse) surface irradiances and outline similarities and differences between the conventional and our method in terms of the aforementioned three steps. An extension of our method to the direct and diffuse components of the total irradiance and their different combinations, such as the DDR (“Different ways of assessing BB events” section), includes three steps outlined above.

**Fig. 8:**
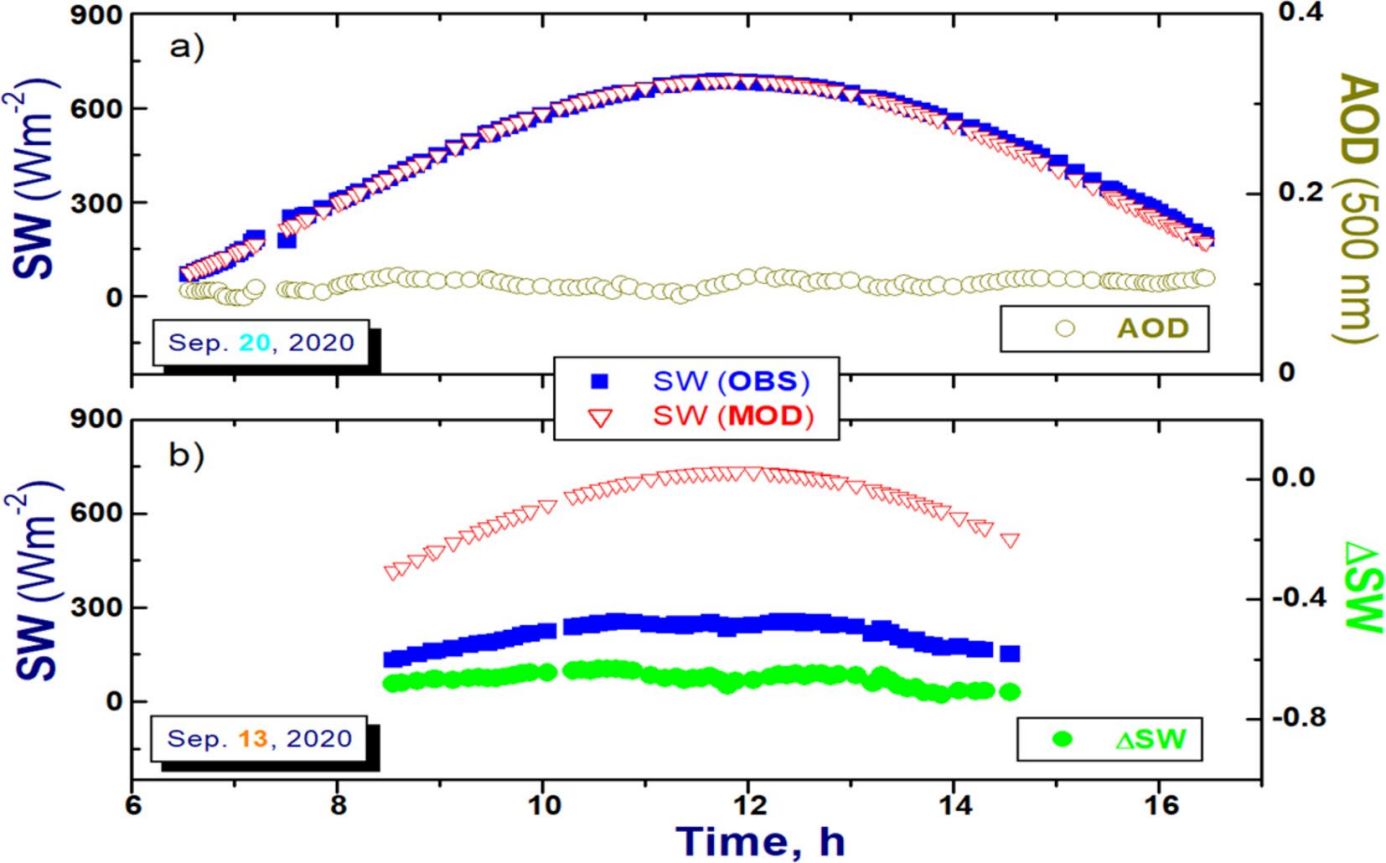
Illustration of the suggested three-step approach: (**a**) selection of the “clean” and “clear-sky” day (September 20, 2020) using AOD measured at 500 nm wavelength (yellow) demonstrates the first step, while the empirical fitting (red; symbol “MOD”) of the measured (blue; symbol “OBS”) SW irradiances for the selected day reveals the second step; (**b**) calculation of the normalized difference (green; symbol ΔSW) using the measured “polluted” (blue) and estimated “clean” (red) SW irradiances for a “polluted” day (September 13, 2020) with smoke plume detected by the ground-based AML instruments explains the third step. Note, the measured AODs are not available in the morning (before 8.5 h; local time) and in the afternoon (after 14.6 h; local time) for this “polluted” day. Thus, the normalized difference is not calculated for these periods.

The first step conveys the main difference between these two methods. The conventional method defines a “clear-sky” day using temporal changes of the direct and diffuse component of the SW irradiance measured at surface without any complementary data^31^. However, there is no assurance that the defined “clear-sky” day can be considered as a “clean”. Our method defines both “clear-sky” and “clean” day using complementary measurements of cloud-screened and quality-assured (Version 3; Level 2.0) AODs provided by a CSPHOT. A large number of these AODs indicates that the ”clear-sky” periods frequently occurred for the intended day, while small values of the daily-averaged (or mean) AOD and its standard deviation suggests “clean” situations. Certainly, wavelength dependent AOD thresholds can be stated for a specific task as needed. For our work, we use the following thresholds for the AOD measured at 500 nm wavelength: moderate-to-large (more than 100) number of AODs and small values (less than 0.1 and 0.01) for the mean and standard deviation, respectively. One day (September 20, 2020) from the selected 12-day period (September 9-20^th^, 2020) meets these AOD-related thresholds: 0.099 (mean), 0.006 (standard deviation) and 109 (number). Thus, we consider this day as both “clear-sky” and “clean” day (Fig. 8a). The main finding of our study is not sensitive to the selected thresholds mainly due to large difference (roughly two orders of magnitude) between “clean” (about 0.1) and “polluted” (about 10) values of AODs at 500 nm wavelength.

The second step is mostly identical for these two methods. Originally, it deals with the empirical fitting of the SW irradiances measured for the “clear-sky” day defined during the first step^31^. We use the same fitting procedure for the SW irradiance (Fig. 8a). In line with the conventional method^31^, the “clean” SW irradiance is estimated using a power law equation: SW_clean_(µ) = Aµ^B^. This equation incorporates the cosine of the solar zenith angle (µ) as the independent variable, along with two fitting coefficients (A and B). The first coefficient (A) indicates the “clean” SW irradiance at a solar zenith angle of 0 degrees and accounts for factors such as average aerosol and column water vapor amounts, the mean Earth-Sun distance on the day being fitted, and the calibration of radiometer. The second coefficient (B) accounts for the cosine response of radiometer. Note that Long and Ackerman (2000) have conducted a thorough evaluation of the accuracy of the estimated "clear-sky" SW fluxes. They specifically demonstrated that neglecting ancillary inputs related to temporal variations in aerosol and water vapor—either throughout the day being fitted or between fitted days—introduces uncertainty into the estimated "clear-sky" SW fluxes^31^. The introduced uncertainty was within the range of the measurement uncertainties themselves^31^. We utilize the same fitting process as Long and Ackerman (2000) to estimate “clean” SW fluxes using supplementary AOD data, thus we expect that the uncertainty of the estimated “clean” SW fluxes will be comparable to the measurement uncertainties. Additionally, we extend the original empirical fitting^31^ to the spectrally resolved surface irradiances. Such extension makes it possible, for instance, to separate the impact of aerosol and surface characteristics on the surface irradiance due to their distinct spectral behaviors.

The third step is mostly identical for these two methods. The conventional method defines the impact of clouds as an *absolute* difference between the measured “cloudy-sky” and estimated “clear-sky” irradiances. Here we define impact of smoke plumes as a *normalized* difference (ΔSW = (SW_polluted_—SW_clean_)/SW_clean_) between the measured “polluted” and estimated “clean” irradiances (Fig. 8b). For our normalization we use the estimated “clean” irradiance. Such normalization aims to eliminate potential observational uncertainties, calibration issues, effects of solar zenith angle and spectral surface albedo α(λ)^37,55^. Recall, the radiative forcing calculations are associated with net fluxes, which are a product of the downwelling surface irradiance and term (1–*α*(*λ*)), and this term is eliminated by normalization. The normalization also makes results obtained for the broadband and spectrally resolved irradiances directly comparable (Fig. 6a). Moreover, it is now possible to contrast the radiative impacts of plumes generated by intense wildfires, dust storms, clouds, and volcanic eruptions. It should be recognized that the “polluted” irradiance can represent partly cloudy observational conditions. To reduce potential cloud contamination on our results, we estimate the radiative impact of smoke plumes only for instances where the cloud-screened and quality-assured (Level 2.0) AODs are available for the “polluted” day. Thus, we use these AODs twice in our approach: (1) for selecting “clear-sky” and “clean” day (the first step) and (2) for screening, at least in part, potential cloud contamination (the third step). The coincident data of surface solar irradiance and AOD allows one to estimate the aerosol radiative forcing efficiency^56–58^.

## Supplementary Information

Supplementary Information.

## Data Availability

Data are available at the following web address: https://data.ess-dive.lbl.gov/view/doi:10.15485/2,335,802.
