# Supplementary material for: Radiative impact of record-breaking wildfires from integrated ground-based data
Source: Sci Rep. 2025 Mar 10;15:8262. doi: 10.1038/s41598-025-85103-1 (PMC11894053; doi:10.1038/s41598-025-85103-1)
Supplement: Supplementary file 1 — Supplementary Information. [file 41598_2025_85103_MOESM1_ESM.docx]

**Radiative impact of record-breaking wildfires from integrated ground-based data**

Evgueni Kassianov^1^, Connor J. Flynn^2^, James C. Barnard^3^, Larry K. Berg^1^, Sherman J. Beus^1^, Xingyuan Chen^1^, Swarup China^1^, Jennifer M. Comstock^1^, Brian D. Ermold^1^, Abdulamid A. Fakoya^2^, Gourihar Kulkarni^1^, Nurun Nahar Lata^1^, Nate G. Mcdowell^1,4^, Victor R. Morris^1^, Mikhail S. Pekour^1^, Joseph Rasmussen^1^, Laura D. Riihimaki^5,6^, Mingjie Shi^1^, Manishkumar B. Shrivastava^1^, Hagen Telg^5,6^, Damao Zhang^1^, Alla Zelenyuk^1^, Damao Zhang^1^

^1^ *Pacific Northwest National Laboratory, Richland, WA, USA*

^2^ *School of Meteorology, University of Oklahoma, Norman, OK, USA*

^3^ *University of Nevada, Reno, NV, USA*

*^4^ School of Biological Sciences, Washington State University, Pullman, WA, USA*

^5^ *Cooperative Institute for Research in the Environmental Sciences, Boulder, CO, USA*

^6^ *National Oceanic and Atmospheric Administration, Global Monitoring Laboratory, Boulder, CO, USA*

**Supplementary Information**

**S1. The AML instrumental suite**

Total and diffuse solar irradiances at five nominal wavelengths (0.415, 0.5, 0.615, 0.673, 0.87 μm) are measured with 20-sec temporal resolution (Fig. S1) by the AML Multifilter Rotating Shadowband Radiometer (MFRSR) with hemispherical receptors that are periodically shaded by rotating bands^1^. These quantities are used to calculate the direct sun irradiance. Before being deployed, the MFRSR head is run through the calibration facility. This includes a standard lamp calibration, cosine response determination, and a mapping of the spectral response function of each filter detector (i.e., the filter function). Once deployed, the instrument is Langley calibrated^2^.


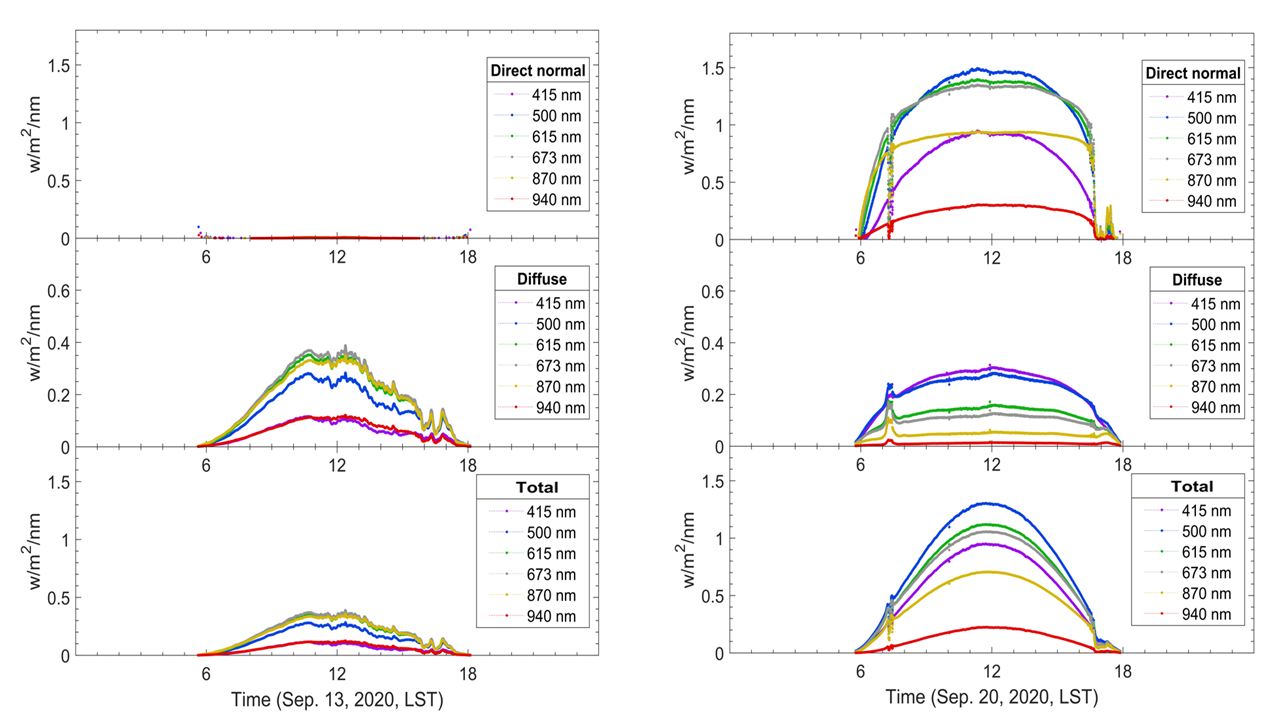


**Figure S1**. Example of diurnal variability of the direct normal (top), diffuse (middle) and total downwelling (bottom) irradiance measured by the AML MFRSR during the “smoky” (September 13, 2020; left panel) and “clean” (September 20, 2020; right panel) days. Horizontal axis is local time.

Downwelling SW (0.3 to 3.0 μm) irradiances are measured with 1-min temporal resolution (Fig. S2, Fig. 5a) by the following three AML instruments^3^: (1) direct normal (beam) irradiance (Fig. S2a) is measured by a pyrheliometer with a 5.7ᵒ field of view (FOV), (2) diffuse horizontal (sky) irradiance (Fig. S2b) is measured by a shaded and ventilated pyranometer with a hemispherical FOV but blocked from the direct normal irradiance by a tracking ball, and (3) total hemispheric (global) irradiance is measured by an unshaded and ventilated pyranometer with a hemispherical FOV (Fig. 5a). Estimated calibration and measurement uncertainties are about ±3.0% for the direct normal (beam) irradiance and about ±4.0% for the diffuse horizontal and total hemispheric irradiances.


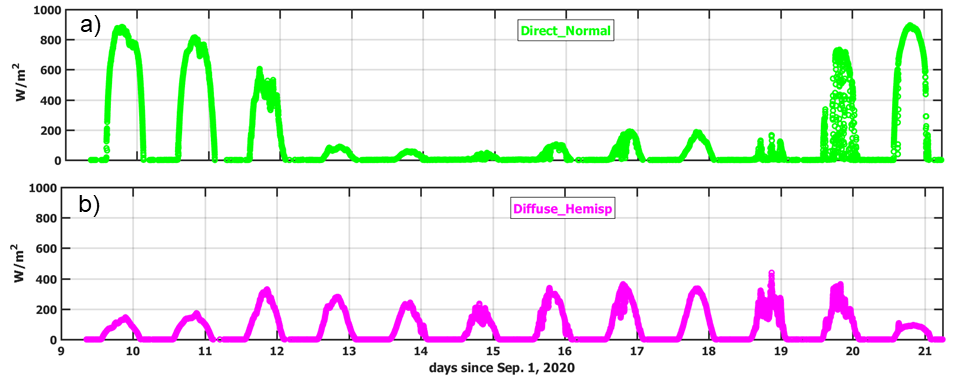


**Figure S2**. Temporal variability of the direct normal SW irradiance (a) and diffuse hemispherical (b) measured by the AML pyrheliometer and pyranometer, respectively. Horizontal axis is time (calendar date in September 2020, UTC).

The AOD at eight wavelengths (0.34, 0.38, 0.44, 0.5, 0.675, 0.87, 1.02, and 1.64 μm) is derived with relatively coarse temporal (about 10–15 min) resolution from the direct sun irradiance. The latter is measured by Cimel Sunphotometers (CSPHOT) with a sun- and sky-tracking design and 1.2° FOV^4^. The calibration uncertainty of AOD is approximately 0.01–0.02. It should be mentioned that the CSPHOT deployed at the AML is part of the National Aeronautics and Space Administration (NASA) Aerosol Robotic Network (AERONET)^5^, and thus AERONET processes the CSPHOT data. The CSPHOT calibration involves intercomparison with reference instruments located at the NASA Goddard Space Flight Center.

Backscatter signals by clouds and aerosols is measured with 10-m vertical resolution by the AML ceilometer. The ceilometer transmits pulses of near-infrared (910 nm) light and the receiver detects the light scattered back by clouds, precipitation, and aerosols through a maximum vertical range of 7700 m^6^. It detects up to three cloud layers simultaneously. The backscatter profile (range and sensitivity normalized) has an estimated uncertainty of ± 0.1*10^-3^ srad^-1^ km^-1^.

Images from the sky are captured via a solid-state charge-coupled device, the so-called Total Sky Imager (TSI), looking downward onto a heated, rotating hemispherical mirror^7^. An opaque blackband on the mirror blocks the intense direct-normal light from the sun, thereby protecting the imager optics. An image-processing algorithm captures and displays the images. The TSI is a daytime imager. Once the sun rises above a user-selectable minimum solar zenith angle, image acquisition begins. TSI images can be used for assessment of fractional sky cover^8-10^, cloud base height^11^, and cloud area^12^.

Air temperature (ambient) at about 5.5 meters above the ground is measured by thermometer exposed to the air in a place sheltered from direct solar radiation, rain, and fog, which can artificially modify it.

**S2. The regional representativeness of the AML instrumental suite**

Figure S3 demonstrates two main points regarding the regional representativeness of the AML site in terms of AOD. First, there is a strong overlap between AOD time series obtained for different spatial footprints when aerosol loading is small-to-moderate (AOD <0.5) (Fig. S3, top panel). It should be emphasized that such aerosol loading is frequent (about 90% of time) over the land^13^. Second, the absolute value of the mean absolute error or deviation (MAE) does not exceed 0.06 (Fig. S3, bottom panel). This value is comparable with expected error envelopes (±0.05 + 15% AOD) of the MODIS-retrieved AOD over land^14^. Similar errors are expected for AOD product offered by the GOES ABI^15^. Note, the analysis presented here is limited to data assessed as high and medium quality, which, for the PNNL region, is available exclusively from the ABI sensor onboard the GOES-17 satellite.

**
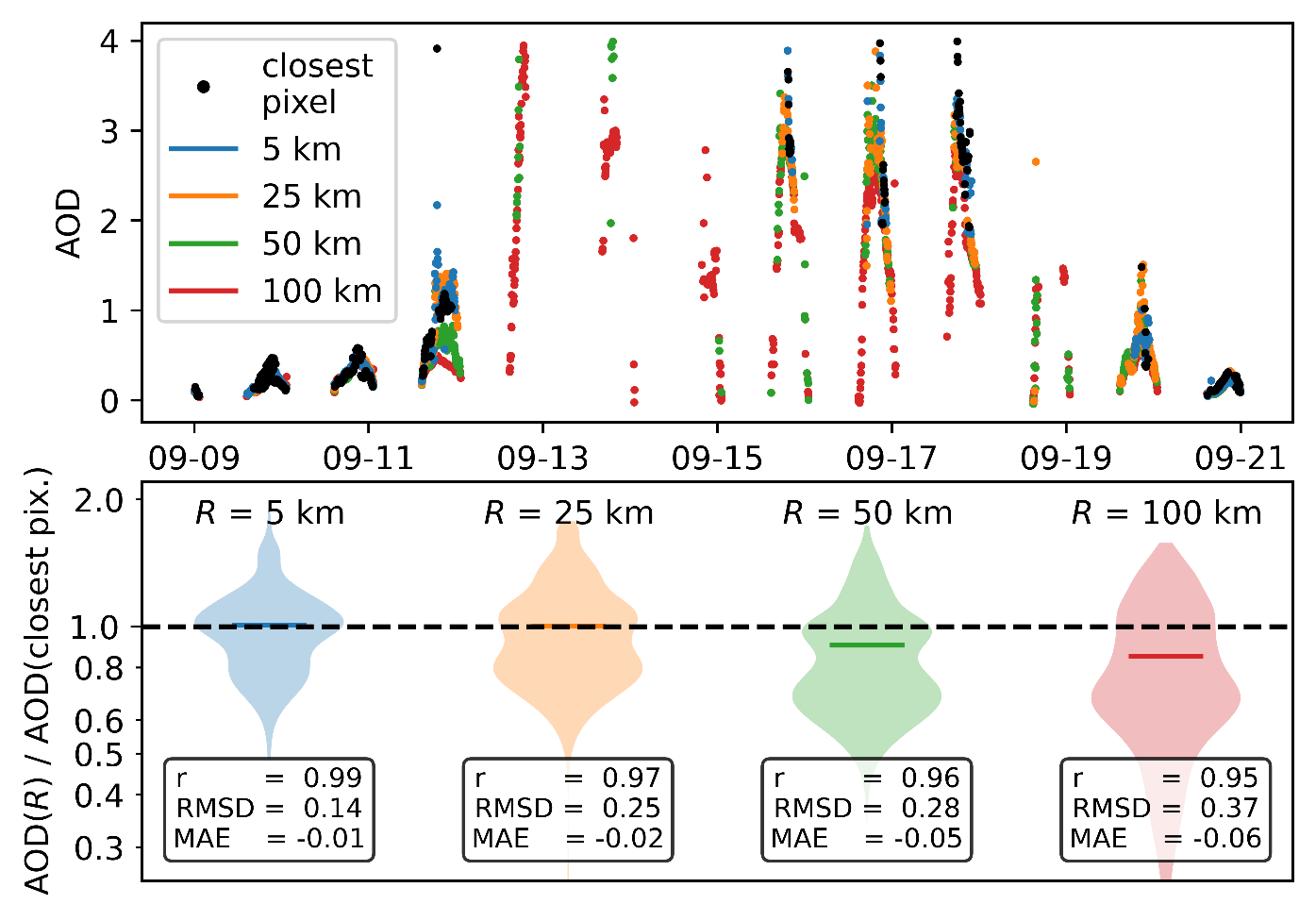
**

**Figure S3**. The upper panel shows AOD values derived at 550 nm wavelength from Advanced Baseline Imager (ABI) sensor observations on the GOES-17 geostationary satellite. These observations have a wide AOD measurement range (up to 5.0) and provide AOD with high temporal (5 min) and spatial (2 km) resolution. The displayed graphs exhibit differing spatial footprints, including values from the pixel nearest to the AML site (AOD(closest pixel)) and the median of all values within specified radii R (5, 25, 50, and 100 km) surrounding the AML site, hereafter referred to as AOD(R). The lower panel of Fig. S3 illustrates the probability distributions of the ratio AOD(R)/AOD(closest pixel) through violin plots, where the probability is denoted by the width of the filled area. Additionally, the lower panel presents the ratio’s median (solid colored line), correlation coefficient r, root-mean-square deviation (RMSD), and mean absolute error or deviation (MAE).

On average, the AOD (closest pixel) collected nearby the AML site effectively defines the areas within a moderate radius (25 km and smaller) in terms of three basis statistics (r, RMSD and MAE) (Fig. S3). As radius R surpasses 25 km, median values between AOD (closest pixel) and AOD(R) progressively deviate. Such deviation is anticipated given the spatial extent of the smoke plume depicted in Fig. 1. Despite the excellent agreement obtained for correlation and median values, there is a substantial variability, as evidenced by the RMSE, even for the smallest area surrounding the site. This variability is likely attributable to the inherent spatial small-scale fluctuations in BB plumes.

**S3. Characterization of ambient aerosol particles over the AML/EMSL**

The biomass-burning smoke particles were sampled from the ambient inlet located about 3 m above the rooftop of the AML building^16^. The particle size distribution was obtained using the TSI laser aerosol spectrometer (LAS; Model 3340A; <https://tsi.com/products/particle-sizers/particle-size-spectrometers/laser-aerosol-spectrometer-3340a/>, last access: 04 May 2023). The LAS provides optical measurements of particles with high temporal resolution (1 Hz) within a wide size range (90-7500 nm). Figure S4 shows the normalized size distributions of smoke and background particles averaged over 24 hours for September 12 (smoky conditions) and September 20 (clean or background conditions). The aerosol size distribution of smoke particles is clearly distinct from the background size distribution in terms of both the mode position and size range (Fig. S4).


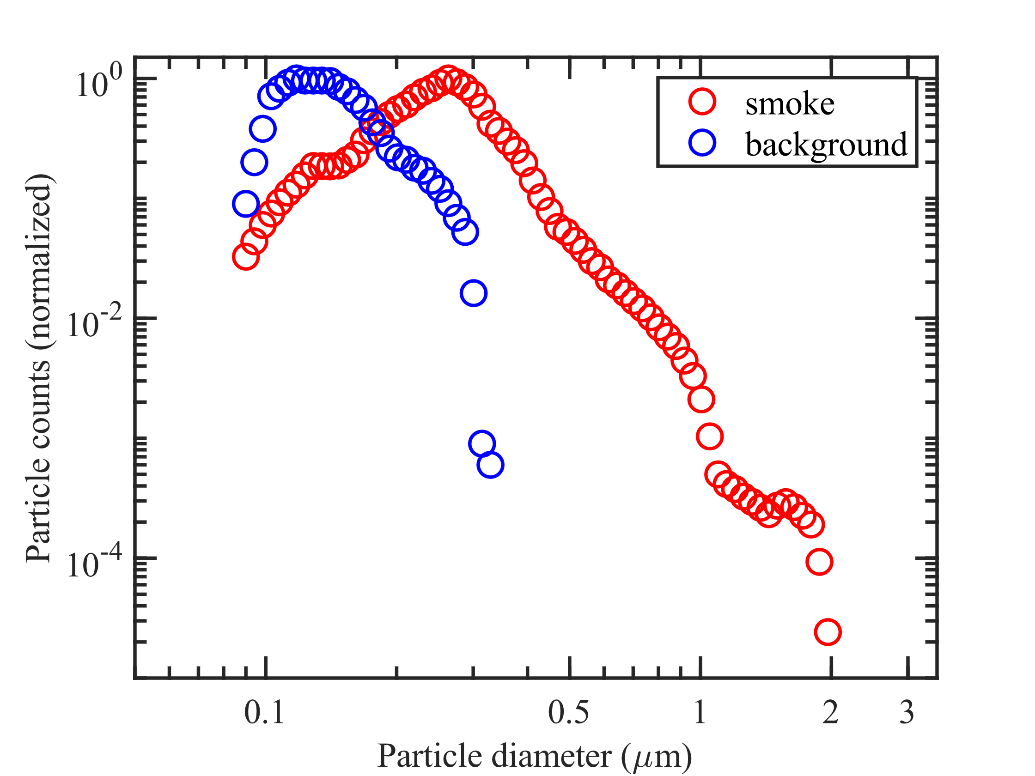


**Figure S4**. The average size distribution normalized by the particle counts of smoke and background particles collected during 12 September 2020 (red) and 20 September 2020 (blue), respectively. The mode diameter of smoke particles is higher than that for background particles. Smoky conditions in comparison with background conditions have a substantial fraction of supermicron (up to 2 µm) particles.

Particles smaller than 1 micron is size (PM1) were sampled from the ambient particulate sharp cut cyclone inlet (Model SCC 1.197, BGI Inc.) installed on the EMSL rooftop. A scanning mobility particle sizer (SMPS, made up of a differential mobility analyzer (DMA, Model 3081, TSI Inc.) and a condensation particle counter (CPC, Model 3786, TSI Inc.) was used for real-time measurements of particle size distributions. PNNL’s single particle mass spectrometer, miniSPLAT^17^, was used for online characterization of single particle size (vacuum aerodynamic diameter) and composition and measurements of composition-resolved particle shape and density^18,19^. Figure S5 presents an example of the SMPS-measured size distribution acquired during period influenced by the biomass-burning events. It shows that, similar to the LAS-measured aerosol size distribution of smoke shown in Figure S4, the particle number concentration peaks at ~300 nm, and drops by an order of magnitude at 0.1 and 0.5 microns. The SMPS-measured size distributions and the miniSPLAT-measured particle density were also used to calculate mass loading of sub-micron aerosols during biomass-burning events, which were found to be nearly 2 orders of magnitude higher compared to the typical background PM1 mass loadings.


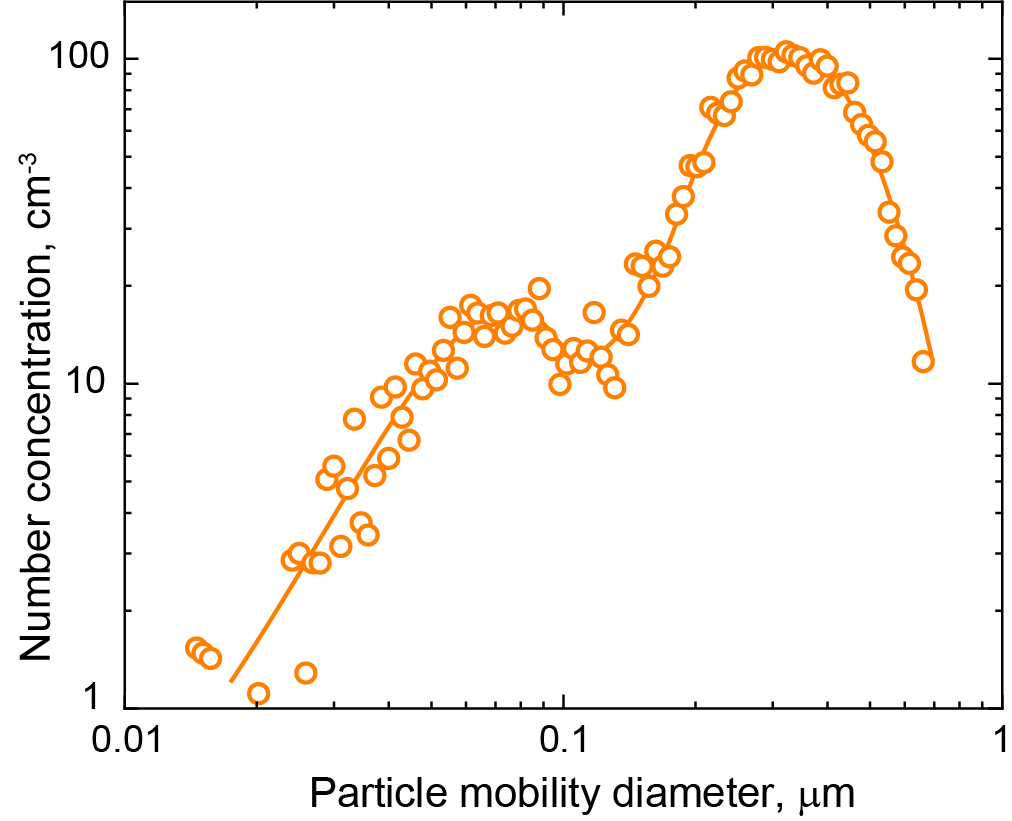


**Figure S5**. Example of particle size distribution measured by the SMPS at EMSL during biomass-burning events.

Figure S6 shows a normalized average mass spectrum of all particles sampled and characterized by miniSPLAT on September 11, 2020. It exhibits mass spectral peaks characteristic of biomass-burning particles (K/K_3_SO_4_) (m/z = 39 and 213) and indicates the presence of organic carbon (OC), including oxidized fragments, e.g., CO, C_2_H_3_O, CO_2_, (m/z = 28, 43, and 44), elemental carbon (EC) (m/z = 12, 24, 36), polycyclic aromatic hydrocarbons (PAHs), some sulfates, and organo-nitrates. The analysis of single particle composition indicates that 88% of particles characterize during this time by miniSPLAT (50% cut-off at 85 nm) were spherical biomass burning particles with density of 1.4 g cm^-3^, while dust particles accounted for 10% of aerosol numbers.


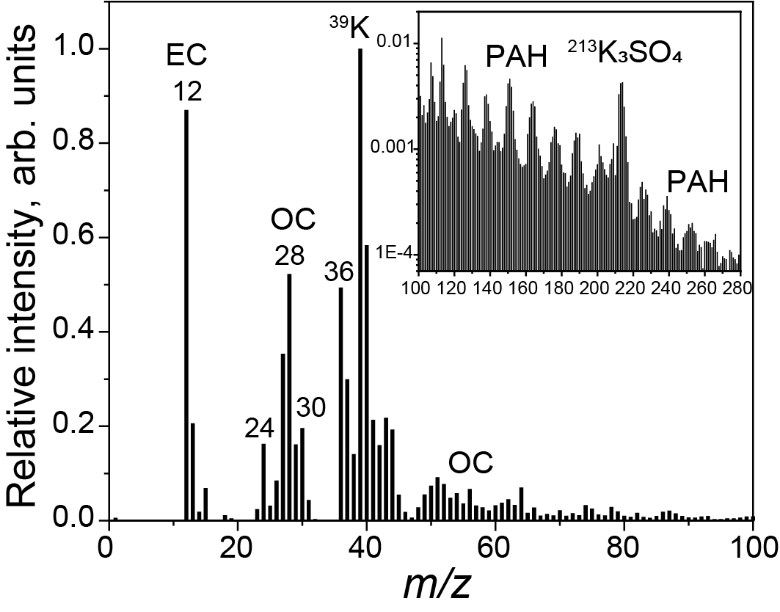


**Figure S6**. An average mass spectrum of biomass burning particles characterized by the miniSPLAT, indicating the presence of EC, OC, including PAHs, and inorganics.

Analysis of individual particles was performed to determine their elemental composition and morphology using computer-controlled scanning electron microscopy with energy-dispersive X-ray spectroscopy (CCSEM/EDX). An environmental scanning electron microscope (Quanta 3D model, Thermo Fisher Scientific) with an EDX spectrometer was used for a seven-day period (September 11-18, 2020). Samples were collected by a 10-stage Micro- Orifice Uniform Deposition Impactors (MOUDI; model 110-R, MSP, Inc.) during the beginning of the “smoky” event (September 11, 2020), while samples were collected by the Sioutas cascade impactor for other days (September 12-18, 2020). Particle samples collected from stages 7 and 8 of MOUDI impactor (50% cut off diameter of 0.32µm and 0.18 µm for stages 7 and 8, respectively) and stage D of Sioutas cascade impactor (50% cut off diameter of 0.25 µm) were used for analysis. Over 14,000 individual particles were examined. These particles were classified based on their elemental compositions as described in detail in previous work^20^.

Figure S7 shows size-resolved chemical composition of particles during smoke plume evolution. Overall, size-resolved chemical composition data show that carbonaceous particles were dominant (Fig. S7). For example, over 95% of particles were carbonaceous on September 11, 2020. The observed dominance is expected for plumes associated with biomass burning. Two interesting features of the plume evolution for the period considered here (September 11-18, 2020) should be highlighted. First, a noticeable fraction of dust, sulfate and sodium-rich particles were observed at the beginning (September 11, 2020) of this period (Fig. S7; top panel). Second, the relative contribution of supermicron particles to the size-resolved chemical composition tends to increase during the plume evolution (Fig. S7; top panel). Such dominance is supported visually by the collected images (Fig. S7; middle and bottom panels).


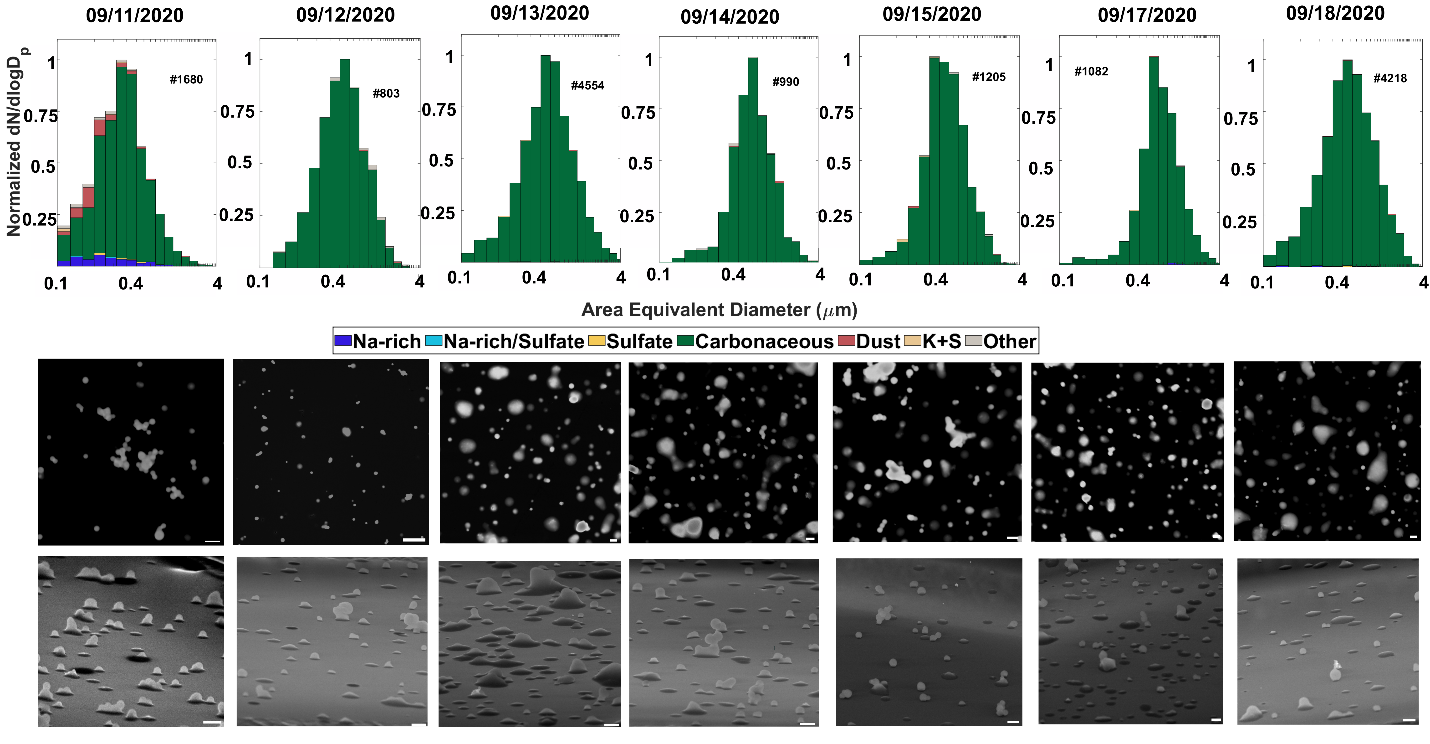


**Figure S7**. Top panel shows size-resolved chemical composition of particles. Middle panel shows top-view and bottom panel shows tilted-view images of particles. Number in the inset shows total number of particles analyzed. All the scale bars in the middle and bottom panels represent 1 µm.

References

1. Hodges, G.B., & Michalsky, J.J. Multifilter Rotating Shadowband Radiometer Instrument Handbook with subsections for derivative instruments: Multifilter Radiometer (MFR) Normal Incidence Multifilter Radiometer (NIMFR). Preprint at https://ww w.arm.gov/publications/tech_reports/handbooks/mfrsr_handbook.pdf (2016)

2. Koontz, A., C,. Flynn, G., Hodges, J., Michalsky, J., Barnard, E., Cromwell, & Kassianov, E. Aerosol Optical Depth Value-Added Product. Preprint at https://www.arm.gov/ publications/tech_reports/doe-sc-arm-tr-129.pdf (2023)

3. Andreas, A., Dooraghi, M., Habte, A., Kutchenreiter, M., Reda, I., & Sengupta, M. Solar Infrared Radiation Station (SIRS) Sky Radiation (SKYRAD) Ground Radiation (GNDRAD) and Broadband Radiometer Station (BRS) Instrument Handbook Preprint at https://www.arm.gov/publications/tech_reports/handbooks/sirs_handbook.pdf (2018)

4. Gregory, L.C. Cimel Sunphotometer (CSPHOT) Handbook Preprint at https://www.arm.gov/publications/tech_reports/handboo ks/csphot_handbook.pdf (2011).

5. Holben, B. N. et al. AERONET – A federated instrument network and data archive for aerosol characterization. Remote Sens. Environ. 66, 1–16 (1998).

6. Morris, V.R. Ceilometer Instrument Handbook. Preprint at https://www.arm.gov/publications/tech_reports/handbooks/ceil_han dbook.pdf (2016).

7. Morris, V.R. Total Sky Imager (TSI) Handbook.U.S. Department of Energy. Preprint at https://www.arm.gov/publications/tech_re ports/handbooks/tsi_handbook.pdf (2005)

8. Long, C.N., & DeLuisi, J.J. Development of an automated hemispheric sky imager for cloud fraction retrievals. In Proceedings 10th Symposium on Meteorological Observations and Instrumentation January 11–16 (Phoenix AZ 1998).

9. Kassianov, E., Long, C. N. & Ovtchinnikov, M. Cloud sky cover versus cloud fraction: whole-sky simulations and observations. J. Appl. Meteorol. 44, 86–98 (2005).

10. Kazantzidis, A., Tzoumanikas, P., Bais, A., Fotopoulos, S. & Economou, G. Cloud detection and classification with the use of whole-sky ground-based images. Atmos. Res. 113, 80–88. https://doi.org/10.1016/j.atmosres.2012.05.005 (2012).

11. Kassianov, E., Long, C. N. & Christy, J. Cloud-base height estimation from paired ground-based hemispherical observations. J. Appl. Meteor. Climatol. 44, 1221–1233 (2005).

12. Kleiss, J. M. et al. Cloud area distributions of shallow cumuli: A new method for ground-based images. Atmosphere 9, 258 (2018).

13. Andrews, E., Ogren, J. A., Kinne, S. & Samset, B. Comparison of AOD, AAOD and column single scattering albedo from AERONET retrievals and in situ profiling measurements. Atmos. Chem. Phys. 17, 6041–6072. https://doi.org/10.5194/acp-17-604 1-2017 (2017).

14. Levy, R. C. et al. The Collection 6 MODIS aerosol products over land and ocean. Atmos. Meas. Tech. 6, 2989–3034 (2013).

15. Huff, A. K. et al. Tracking smoke from a prescribed fire and its impacts on local air quality using temporally resolved GOES-16 ABI aerosol optical depth (AOD). J. Atmos. Oceanic Technol. 38, 963–976. https://doi.org/10.1175/JTECH-D-20-0162.1 (2021).

16. Kulkarni, G. et al. A new method for operating a continuous-flow diffusion chamber to investigate immersion freezing: assessment and performance study. Atmos. Meas. Tech. 13, 6631–6643. https://doi.org/10.5194/amt-13-6631-2020 (2020).

17. Zelenyuk, A. et al. Airborne single particle mass spectrometers (SPLAT II & miniSPLAT) and new software for data visualization and analysis in a geo-spatial context. J. American Soc. for Mass Spectrometr. 26, 257–270 (2015).

18. Zelenyuk, A., Yang, J., Song, C., Zaveri, R. A. & Imre, D. A new real-time method for determining particles’ sphericity and density: application to secondary organic aerosol formed by ozonolysis of alpha-pinene. Environ. Sci. Technol. 42, 8033–8038 (2008).

19. Vaden, T. D., Imre, D., Beranek, J. & Zelenyuk, A. Extending the capabilities of single particle mass spectrometry: II Measurements of aerosol particle density without DMA. Aerosol Sci. Technol. 45, 125–135 (2011).

20. Lata, N. N. et al. Aerosol composition, mixing state, and phase state of free tropospheric particles and their role in ice cloud formation. ACS earth and space Chem. 5, 3499–3510. https://doi.org/10.1021/acsearthspacechem.1c00315 (2021).
